# RHIVDB: A Freely Accessible Database of HIV Amino Acid Sequences and Clinical Data of Infected Patients

**DOI:** 10.3389/fgene.2021.679029

**Published:** 2021-06-10

**Authors:** Olga Tarasova, Anastasia Rudik, Dmitry Kireev, Vladimir Poroikov

**Affiliations:** ^1^Department of Bioinformatics, Institute of Biomedical Chemistry, Moscow, Russia; ^2^Central Research Institute of Epidemiology, Moscow, Russia

**Keywords:** antiretroviral therapy, drug exposure, therapy success, database, human immunodeficiency virus, HIV, sequence data analysis, HIV drug resistance

## Abstract

Human immunodeficiency virus (HIV) infection remains one of the most severe problems for humanity, particularly due to the development of HIV resistance. To evaluate an association between viral sequence data and drug combinations and to estimate an effect of a particular drug combination on the treatment results, collection of the most representative drug combinations used to cure HIV and the biological data on amino acid sequences of HIV proteins is essential. We have created a new, freely available web database containing 1,651 amino acid sequences of HIV structural proteins [reverse transcriptase (RT), protease (PR), integrase (IN), and envelope protein (ENV)], treatment history information, and CD4+ cell count and viral load data available by the user’s query. Additionally, the biological data on new HIV sequences and treatment data can be stored in the database by any user followed by an expert’s verification. The database is available on the web at http://www.way2drug.com/rhivdb.

## Introduction

Human immunodeficiency virus (HIV) along with other viruses has a high social impact due its ability to spread from one person to another. According to the latest data^1^, in 2020, the estimated number of new infection cases was over 1.5 million, while more than 38 million people are currently living with HIV [see text footnote 1]. All known antiretroviral drugs can only suppress viral replication but it is still impossible to eliminate the virus from human body completely (Geronikaki et al., 2016). Due to its high mutagenicity HIV is capable to develop resistance, to existing antiretroviral drugs (Geronikaki et al., 2016). Data on the amino acid sequences of HIV proteins, including reverse transcriptase (RT), protease (PR), integrase (IN), and envelope protein (ENV), are important for the prediction of HIV drug resistance (Liu and Shafer, 2006; Toor et al., 2011; Raposo and Nobre, 2017; Ramon et al., 2019; Steiner et al., 2020) and the so-called drug exposure, which is considered one of the features potentially associated with HIV drug resistance (Pironti et al., 2017). With data from the (i) amino acid sequences of HIV proteins, (ii) drug combinations used to treat HIV-positive patients, and (iii) clinical data obtained from the patients, it is possible to build models predicting (a) drug exposure and HIV drug resistance and (b) therapeutic effectiveness based on the HIV sequence data and the treatment history (Tarasova et al., 2020).

There are databases of amino acid and nucleotide sequences of HIV freely available for downloading and analysis (Kuiken et al., 2003; Rhee et al., 2003; Shafer, 2006). Particularly, Los Alamos National Laboratory (LANL) HIV sequence database contains over 900,000 sequences of HIV, which can be found by a user’s query. Retrieved sequences can be aligned to assess their similarity with resistant samples or to investigate phylogeny. LANL HIV sequence database also contains premade alignments that can be used to investigate frequently occurred mutations, which may cause drug resistance. HIV drug resistance database (Rhee et al., 2003), developed and maintained at Stanford University, includes three main types of data: “genotype-phenotype,” “genotype-treatment,” and “genotype-clinical.” “Genotype-phenotype” relationship includes information about HIV sequences and the data on their drug resistance/susceptibility, including resistance against HIV RT, PR, and IN inhibitors. It includes data on over 15,000 isolates tested on drug resistance in various assays. “Genotype-treatment” data includes over 300,000 sequences retrieved from HIV samples with the set of drugs taken by a patient. “Genotype-clinical” data contains over 1,500 episodes of the particular drug combinations taken by a patient along with some clinical data (CD4+ cell count and viral load at the time). There are statistics on the mutation prevalence, patterns of drug resistance mutations, and a summary of major and minor drug resistance positions. These databases are beneficial for HIV drug resistance analysis.

In addition to the databases that have already been developed, we have created a new, freely available web database, RHIVDB, to provide comprehensive data on HIV amino acid sequences, clinical data, and drug treatment history information. The main feature of RHIVDB is the availability of drug treatment history and clinical data for each record.

RHIVDB is developed based on the clinical data and the data on amino acid sequences of the HIV proteins collected in the Russian Federation in the Central Research Institute of Epidemiology. The database contains information about amino acid sequences of HIV proteins, drug combinations that were taken by a patient during a particular period, and CD4+ cell count and viral load data available for fast downloading on the user’s query. The database can be used for determining the effectiveness of particular drug combinations, analysis of HIV sequences for various cohorts of patients, building models for prediction of therapeutic success based on sequence, clinical, and drug history data.

## Methods

Plasma samples were obtained as part of routine drug resistance testing in all federal districts of the Russian Federation. The dates of diagnosis include years from 1997 to 2019. Blood sampling dates ranged from January 2014 to December 2019.

RNA extraction and HIV genome amplification were carried out by ViroSeq HIV-1 Genotyping System (Abbott Molecular, United States) or AmpliSens HIV-Resist-Seq (Central Research Institute of Epidemiology, the Russian Federation). The amplified region of the pol region was at least 1,092 nucleotides length and covered positions 2,253–3,344 with respect to the reference HIV-1 strain HXB2 [GenBank: K03455 (Ratner et al., 1985)]^2^. The amplified region of the env region was 420 nucleotides length and covered positions 6,954–7,374 of HXB2 strain. The nucleotide sequences of the pol and env regions were obtained using Sanger sequencing. Nucleotide sequences represented the part of the pol region encoding HIV PR and RT. Therefore amino acid sequences obtained from nucleotide sequences include corresponding PR and RT parts.

Data on HIV sequences with drug combinations used and data on CD4+ and viral load titer were processed for (i) duplicates removal; (ii) standardization of the drug names representation; (iii) verification of amino acid sequences data.

**FIGURE 1:**
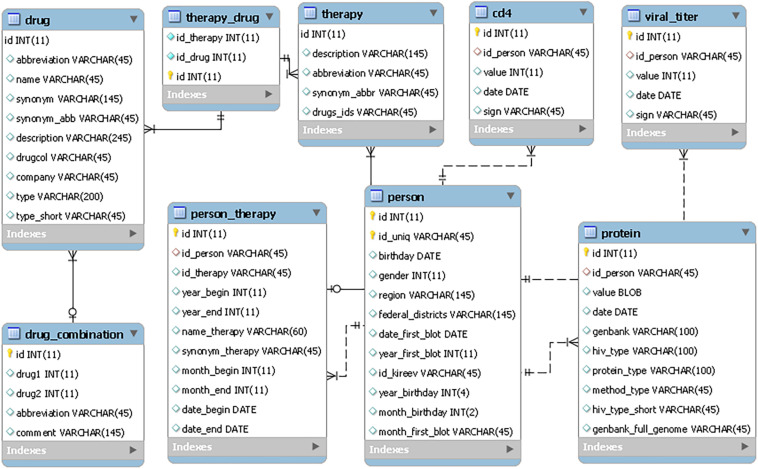
The RHIVDB database schema.

The RHIVDB web database uses the MySQL server to store data. The schema of the database is provided in Figure 1.

PHP and HTML codes were used to implement the main interface, and jQuery plugin DataTables for data accessing and manipulating (sorting, paging, and filtering). The scripts for data export and search were developed using PHP scripting language. They are available as a part of Supporting Information.

## Results

The RHIVDB database contains data on the amino acid sequences of HIV proteins, including the RT, PR, integrase IN, and HIV envelope proteins. In addition, it includes combinations of antiretroviral drugs taken during a particular time period and CD4+ cell count and viral load data during the periods of treatment in the database. The data stored in the RHIVDB do not contain any personal information about patients. The database is freely available on the web [see text footnote 3].

As of March 2021, the database contains 1,653 records on HIV-1 sequence data collected from different patients. For the web-accessible database, we chose only the records that consisted of both sequence and clinical data. For all 1,094 patients, there is information about CD4+ cell count and the number of HIV RNA copies per one ml. Sequence data on RT and PR are available for all 1,094 patients, while for IN and ENV, the data on sequences are available for 281 and 276 patients, respectively. For 434 records, there are data on the drug combinations taken by patients. The total numbers of records corresponding to each parameter are shown in Table 1.

**TABLE 1:** Database characteristics. Number of records and values for each quantitative parameter of the database (A); number of records containing data on drug combinations and viral sequences (B).

(A)			

Parameter	Number of records	Mean	Standard deviation
CD4+ cell count	1,732	343.3	266
Viral load (copies per ml)	1,823	62,475	57,625
Age	1,093	39	9.96

**(B)**			

**Parameter**	**Number of records**		

HIV RT and PR amino acid sequences	1,653		
HIV IN amino acid sequences	281		
HIV ENV amino acid sequences	276		
Drug combination, total	434		
Protease inhibitors	104		
Reverse transcriptase inhibitors (NRTIs)	409		
Reverse transcriptase inhibitors (NNRTIs)	344		
Integrase inhibitors	31		

The database interface provides data on therapy, with the periods during which the particular drug or a combination was taken, and the flag indicating therapy change during treatment. CD4+ cell count and viral load parameters measured in a certain data are provided in the columns “CD4” and “Viral load.” The database includes the patient’s information about age, gender, the date of diagnosis.

The user can perform a search using keywords (“Search” tab). Complex queries are available through the Filter option. It is possible to include several simple queries and combine them using Boolean operators “and”, “or.” Additionally, a user can quickly examine the records satisfying a particular CD4+ cell count or viral load (titer). Retrieved data can be easily exported in Microsoft Excel (CSV), Adobe Acrobat Reader (PDF), and Extensible Markup Language (XML) formats by selecting a particular option. Such options provide an easy way to process the data stored in the database.

Contributions to the database are possible for registered users who are signed in. After data verification by the experts, the information can be added into the database.

If antiviral drug resistance occurs, it is necessary to change a patient’s antiretroviral therapy. On average, for each patient from the database, there are two schemas of therapy. The maximum number of therapy regimens per person is 14. The data stored in the database allow the user to collect information about the therapy (a drug combination) and its effects on the viral load and CD4+ cell count.

## Discussion

The RHIVDB database information provides basis for the selection of the most effective treatment schema and for building models of treatment effectiveness based on clinical data (CD4+ cell count, viral load). The data on the amino acid sequences can be used along with treatment and clinical data to predict drug exposure or treatment effectiveness (Tarasova et al., 2020).

**FIGURE 2:**
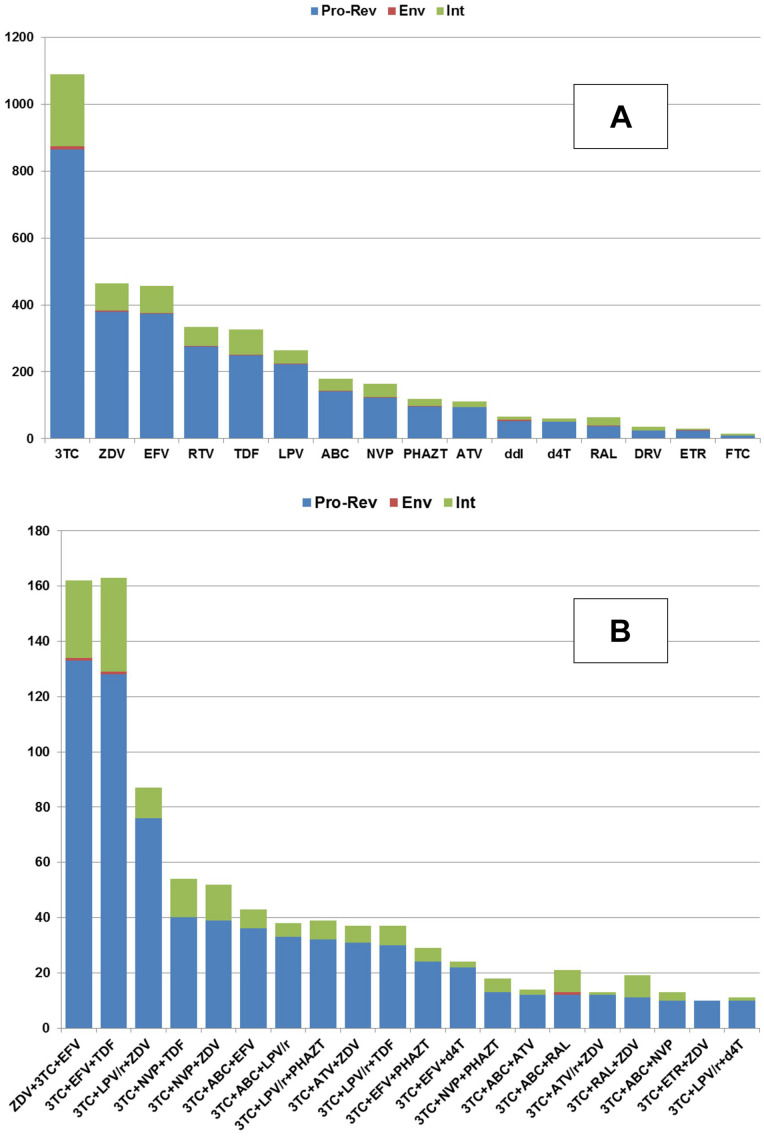
The availability of the amino acid sequences (Y-axis) collected for each HIV protein in association with the particular amino acid sequences obtained from the patients taking a particular drug **(A)** or drug combination **(B)**.

The correlations of the number of HIV-1 sequence to antiretroviral drug combination (A) and to the individual drugs (B) that the patient was taking before the sequence was determined are shown in Figure 2.

The database can help evaluate the therapeutic effectiveness and estimate the mutations’ occurrence related to a patient’s particular drug or drug combinations. Further, we demonstrate its applicability for two purposes: (i) search for CD4+ count and viral titer for particular drug combinations and (ii) evaluating the mutation frequency associated with nucleoside inhibitor abacavir as a case study.

**FIGURE 3:**
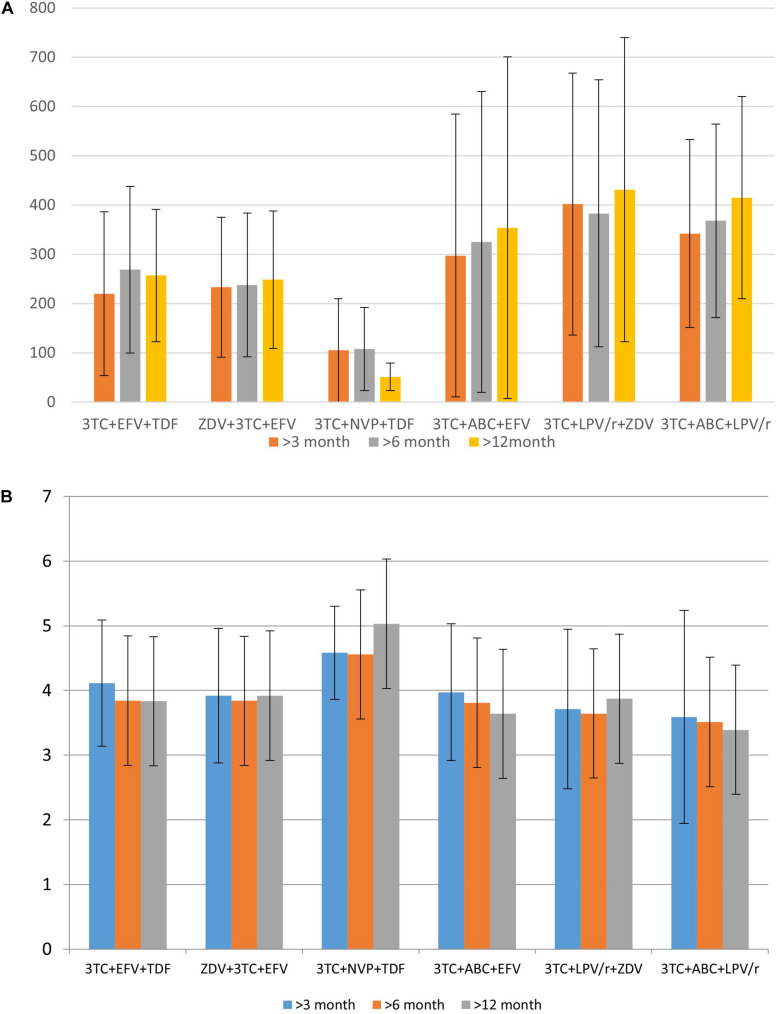
The distribution of mean CD4+ cell count **(A)** and logarithmic values of viral load **(B)** for specific drug combination.

Based on the data collected in the database, it is possible to identify some associations between drugs taken by a patient and CD4+ lymphocytes count or viral load. These parameters, along with the clinical symptoms, are used for the understanding of therapeutic success. We illustrate the applicability of the database for such purposes. Figures 3A,B display the distribution of the mean CD4+ cell count and various viral load values for specific drug combinations, respectively.

Data in Figure 3 provides information regarding drug combinations characterized by the highest and lowest therapeutic efficacy for the cases included in the database. Additionally, in most cases, the average viral load values are remarkably similar to each other after 3 months, 6 months, and 1 year after the beginning of therapy; the same trend might be observed and for CD4+ cell count. It means that in most cases if a drug combination is effective 3 months after the beginning of the therapy, there is a high chance that it is effective after a year.

To demonstrate the applicability of the database to the estimation of amino acid substitutions prevalence associated with a particular drug, we performed such analysis for abacavir and zidovudine (nucleoside reverse transcriptase inhibitors) as a case study. We performed the search in the database and selected 101 and 247 amino acid sequences associated with abacavir and zidovudine taken by a patient, respectively. Based on the dates of therapy changes, we selected 39 sequences, for which the period of therapy with abacavir exceeded 90 days. The number of sequences associated with zidovudine (for the period over 90 days) was 245. If an exact date of therapy change is unknown, sequences obtained in the same year were excluded. These sequences were aligned using the ClustalW tool (Sievers et al., 2011). As a result, we obtained a set of substitutions associated with therapy schemes included abacavir and zidovudine (Table 2).

**TABLE 2:** The substitutions appeared in the amino acid sequences of HIV RT of the samples, retrieved from patients that used nucleoside RT inhibitor abacavir or zidovudine in therapy schemes.

		Abacavir		Zidovudine	
Position	Substitution	N	%	N	%
35	T	23	59	158	64
	I	9	23	59	24
	A (R)	5	2	2	0.8
	**V**	2	5	19	7
	M	2	5	3	2
	K	2	5	4	2
36	D	22	56	88	36
	**E**	20	51	157	64
43	**K**	34	87	234	96
	E	2	5	6	2
	A	1	3	2	0.8
	R	2	5	3	1
49	**K**	36	92	239	97
	R	3	8	6	3
60	V	30	77	218	88
	**I**	9	23	27	12
65	**K**	33	85	195	79
	R	4	10	50	21
	N	2	5	0	0
70	**K**	35	90	232	94
	R	4	10	10	5
	E	0	0	2	0.8
	G	0	0	1	0.2
74	**L**	31	79	230	93
	V	17	44	10	5
90	**V**	33	85	215	87
	I	6	15	30	13
101	**K**	27	69	188	76
	E	7	18	55	23
	Q	4	10	0	0
	R			2	0.8
103	**K**	34	87	217	89
	N	5	13	28	11

*The amino acid residue of the consensus HIV reverse transcriptase sequence is provided in bold.*

It is worth noting that some of them are included in the list of major drug mutations associated with therapy schemes included nucleoside reverse transcriptase inhibitors (for instance, 65 K/R, 74 V/L)^3^, while other substitutions are not common. Interestingly, some of these sequences are characterized by substitutions at 101 and 103 positions, typically associated with resistance to NNRTIs. This example demonstrates that using RHIVDB it is possible to obtain some new information about substitutions that can be associated with the particular drug taken as a part of therapeutic drug combinations.

The further development of our database will provide an opportunity to collect data on various groups of patients who may have different susceptibilities to HIV infection (Lieberman et al., 2001; Jülg and Goebel, 2005; Gonzalo-Gil et al., 2017; Pironti et al., 2017; Lopez-Galindez et al., 2019; Ivanov et al., 2020). We believe that RHIVDB will help analyzing information about patients who do not develop a high viral load over a long time period. The information about the patients, sequence data, CD4+ cell count, and viral load may be used for developing the models of viremic control based on the patients’ data and viral sequences. Therefore, the database can be helpful for developing personalized methods for HIV/AIDS treatment. These methods in particular may include the analysis of gene expression of the HIV-positive patients, analysis of a therapy regimen, allowing identify their individual reply to the particular combination of antiretroviral drugs.

## Conclusion

We developed the database of HIV amino acid sequences containing the data on the combinations of antiretroviral therapy taken by a patient. Additionally, it contains information on the blood parameters that indicate the severity of HIV infection progress and the effectiveness of antiretroviral drug therapy. RHIVDB can be used by clinical specialists, biologists, bioinformatics for analysis of therapy effectiveness, HIV susceptibility and its resistance to antiretroviral therapy, and the variability of HIV sequences considering drug therapy. This database is available on the Internet for any user, it does not require registering an account. The biological data on new HIV sequences and data of therapy can be stored in the database by any user followed by the verification by an expert in the field of HIV epidemiology.

## Data Availability Statement

The datasets presented in this study can be found in online repositories. The names of the repository/repositories and accession number(s) can be found below: Dataryad and accession name RHIVDB data. https://datadryad.org/stash; https://datadryad.org/stash/share/iqb_UwuHd61_I5_z6bot9ui9PKoeEzBxpxX187vnEy0.

## Author Contributions

OT: idea, manuscript writing, and review. AR: database realization and manuscript writing. DK: collecting amino acid sequences, clinical data on CD4+ cell count, viral load, and manuscript editing. VP: manuscript review and editing. All authors contributed to the article and approved the submitted version.

## Conflict of Interest

The authors declare that the research was conducted in the absence of any commercial or financial relationships that could be construed as a potential conflict of interest.

## Footnotes

1 http://www.who.int

2 https://www.ncbi.nlm.nih.gov/nuccore/1906382

3 NRTI Resistance Notes – HIV Drug Resistance Database (https://www.stanford.edu/)
